# Lymphangioma circumscriptum of the scrotum: Case report

**DOI:** 10.1016/j.amsu.2021.102962

**Published:** 2021-10-15

**Authors:** Sara Bouabdella, Siham Dikhaye, Nada Zizi

**Affiliations:** a Department of Dermatology, Mohammed the VIth University Hospital of Oujda, Morocco; b Laboratory of Epidemiology, Clinical Research and Public Health, Faculty of Medicine and Pharmacy, Mohamed the First University of Oujda, Morocco

**Keywords:** Lymphangioma circumscriptum, Scrotum, Lymphatic malformation

## Abstract

**Introduction and importance:**

Lymphangioma circumscriptum (LC) is the most common form of cutaneous lymphangioma. However, scrotal LC is rare.

**Case presentation:**

We report a case of a 37-year-old patient with a complaint of a slow growing painless scrotal grouped lesions present for the past ten years diagnosed as LC.

**Clinical discussion:**

The disorder is clinically identified by translucent or hazy vesicles of different sizes which are grouped like frog spawn or, less commonly, as diffuse swelling to a particular area. The definitive diagnosis is usually made by biopsy. LC tends to be asymptomatic. However, it can be complicated. The treatment involves medical and surgical procedures.

**Conclusion:**

Scrotal LC is rare and tends to mimic certain infectious diseases. The clinicians should be aware of LC in adult males without a prior disease to avoid missing the diagnosis and to prevent inappropriate treatment.

## Highlights

• Lymphangioma circumscriptum is a rare benign skin disorder involving hamartomatous lymphatic malformation.
• Scrotal Lymphangioma circumscriptum is very rare.
• It may present as a congenital condition or, rarely, might develop secondary to radiotherapy, infection, or surgery.

## 1 Introduction

Lymphangioma circumscriptum (LC) is a rare benign skin disorder involving hamartomatous lymphatic malformation of deep dermal and subcutaneous lymphatic channels [1]. Scrotal LC is very rare and may present as a congenital condition or, rarely, might develop secondary to radiotherapy, infection, or surgery [1]. It may be a diagnostic challenge when appearing in the anogenital region and often is misdiagnosed and mistreated as infectious etiologies [2]. We report a healthy 37-year-old man with a ten-year history of an eruption of multiple, asymptomatic, scrotal papules that were diagnosed as lymphangiomas. Our case report was written according to CARE guidelines [3].

## 2 Case presentation

**Fig. 1:**
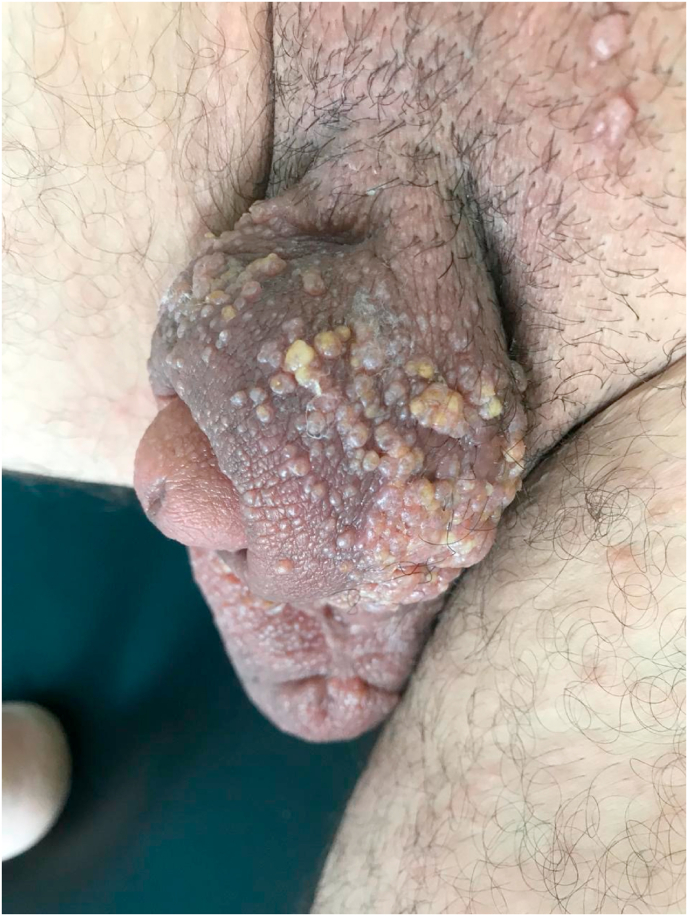
Clinical image: multiple grouped vesicles on the scrotal skin.

**Fig. 2:**
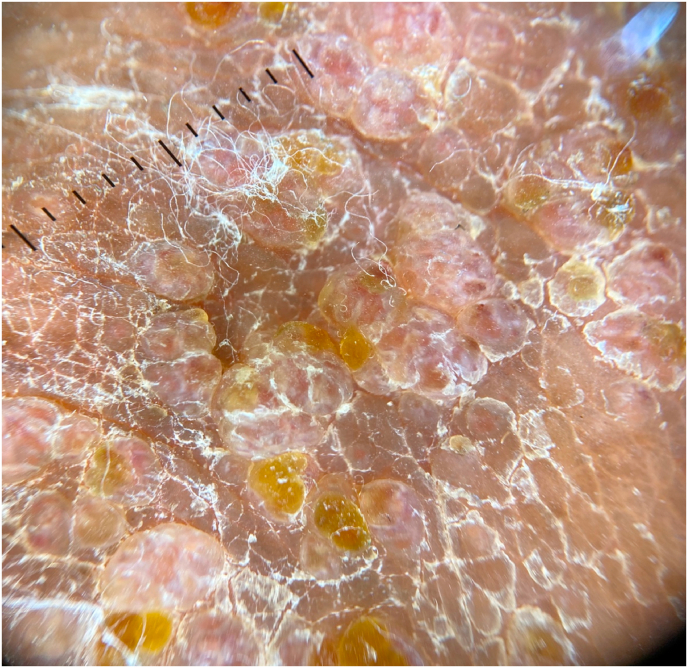
Dermoscopic image showing multiple, grouped flesh-colored, slightly erythematous nodules on the scrotal skin along with several translucent lacunae with a yellow hue; some nodules showed linear vessels. (For interpretation of the references to color in this figure legend, the reader is referred to the Web version of this article.)

A 37-year-old man presented to our department with a complaint of a slow growing painless scrotal grouped lesions present for the past ten years. There was no history of fever or weight loss. Also, there was no similar cases in the patient's family. On examination multiple grouped vesicles were seen involving almost the complete scrotal skin (Fig. 1). Dermoscopy revealed multiple, grouped flesh-colored, slightly erythematous nodules on the scrotal skin along with several translucent lacunae with a yellow hue; some nodules showed linear vessels (Fig. 2). He had no significant past medical history of sexually transmitted disease or surgical procedure in that area. He had a normal laboratory checkup including blood biochemistry, serum lactate dehydrogenase levels and serology for sexually transmitted diseases. There was no peripheral eosinophilia. Detailed physical examination, serology and the absence of eosinophilia excluded the possibility of the common etiology, filariasis. Differential diagnoses included also squamous cell carcinoma, papillomatous carcinoma or a lymphangiosarcoma. A skin biopsy was performed, and the specimen was sent for histopathology. The histopathological examination revealed a very rare disorder of the scrotum with dilated thin-walled lymphatic channels just beneath the skin. There was no endothelial swelling granulomatous reaction or adult worm in the section examined and a diagnosis of lymphangioma circumscriptum was made. A scrotal ultrasound was performed and had shown a phlegmonous infiltration of the scrotal cutaneous and subcutaneous soft tissues, without individualization of a clear collection with a bilateral hydrocele. Body scan was normal. Treatment with Everolimus was indicated but refused by the patient. He was lost to follow-up and did not respond to our telephone calls.

## 3 Discussion

Lymphangioma circumscriptum is the most common form of cutaneous lymphangioma. It constitutes 4% of all vascular tumors. Although it is known to be a congenital lymphatic malformation, there are reports of acquired lymphangioma circumscriptum occurring in areas of chronic lymphedema [4]. It can occur anywhere in the skin and mucous membranes, the common sites being axillary folds, shoulders, neck, proximal part of the limbs, tongue, vulva, buccal mucus membrane and uncommonly the scrotum [5]. Whimster first proposed the pathogenesis of LC in 1976. He postulated that LC is a collection of subcutaneous lymph cisterns, which arise during embryonic development, that are not connected to the lymphatic system and therefore unable to drain the lymph received from surrounding tissue. The cisterns are lined with muscle that contracts and, by applying pressure, produces protrusions on the skin [4].The disorder is clinically identified by translucent or hazy vesicles of different sizes which are grouped like frog spawn or, less commonly, as diffuse swelling to a particular area [5]. Dermoscopy shows two distinct patterns: yellow lacunae surrounded by pale septa, and reddish-blue lacunae with pale septae [6]. The reddish-blue color occurs due to the presence of blood within the lacunae, which might sediment in the bottom in some lacunae, giving the lesions a hypopyon-like appearance. This feature of half-and-half lacunae has been described to differentiate LC from other vascular lesions, such as hemangioma [6]. Clinically, differential diagnosis of cutaneous LC are other vascular lesions, such as hemangioma, angiokeratoma, warts, molluscum contagiosum, and epidermal nevi [6]. The definitive diagnosis is usually made by biopsy. Histopathology of LC revealed dilated lymph vessels with a lining of flat endothelial cells, mostly in the upper dermis and subcutis connected with deeper subcutaneous lymphatic cisterns [1]. Ultra-sonography and CT scan of the abdomen or pelvis are helpful in patients who have suspicious extensions of cystic lesion to the retroperitoneum or pelvis [5]. MRI helps to determine the depth and extent of involvement of LC, which can be extensive [6]. Lymphangioma circumscriptum tends to be asymptomatic. However, it can be complicated by hemorrhage, swelling, bruising and recurrent cellulitis [4]. The treatment of choice for LC is surgical excision; however, less invasive methods are also used. It includes imiquimod, everolimus, sirolimus, bleomycin, cryotherapy, sclerotherapy and radiofrequency coagulation. Ablative CO2 lasers, and suction lipectomy have also been used with variable results and recurrences [6].

## 4 Conclusion

Scrotal LC is rare and tends to mimic certain infectious diseases. The clinicians should be aware of LC in adult males without a prior disease to avoid missing the diagnosis and to prevent inappropriate treatment.

## Consent

Written informed consent was obtained from the patient for publication of this case report. CARE guidelines were applied for reporting this case report’ finding.

## Ethical approval

The ethical committee approval was not required give the article type (case report).However, the written consent to publish the clinical data of the patients was given and is available to check by the handling editor if needed.

## Sources of funding

None.

## Author contribution

Sara Bouabdella: Study concept, Data collection, Data analysis, Writing the paper.

Siham Dikhaye: Supervision and data validation.

Nada Zizi: Supervision and data validation.

## Consent

Written informed consent was obtained from the patient for publication of this case report and accompanying images. A copy of the written consent is available for review by the Editor-in-Chief of this journal on request.

## Research registration

This is not an original research project involving human participants in an interventional or an observational study but a case report. This registration is was not required.

## Guarantor

Sara Bouabdella.

## Provenance and peer review

Not commissioned, externally peer-reviewed.

## Declaration of competing interest

None.
